# Mechanisms of Myofascial Pain

**DOI:** 10.1155/2014/523924

**Published:** 2014-08-18

**Authors:** M. Saleet Jafri

**Affiliations:** Krasnow Institute for Advanced Study, George Mason University, 4400 University Drive, MNS 2A1, Fairfax, VA 22030, USA

## Abstract

Myofascial pain syndrome is an important health problem. It affects a majority of the general population, impairs mobility, causes pain, and reduces the overall sense of well-being. Underlying this syndrome is the existence of painful taut bands of muscle that contain discrete, hypersensitive foci called myofascial trigger points. In spite of the significant impact on public health, a clear mechanistic understanding of the disorder does not exist. This is likely due to the complex nature of the disorder which involves the integration of cellular signaling, excitation-contraction coupling, neuromuscular inputs, local circulation, and energy metabolism. The difficulties are further exacerbated by the lack of an animal model for myofascial pain to test mechanistic hypothesis. In this review, current theories for myofascial pain are presented and their relative strengths and weaknesses are discussed. Based on new findings linking mechanoactivation of reactive oxygen species signaling to destabilized calcium signaling, we put forth a novel mechanistic hypothesis for the initiation and maintenance of myofascial trigger points. It is hoped that this lays a new foundation for understanding myofascial pain syndrome and how current therapies work, and gives key insights that will lead to the improvement of therapies for its treatment.

## 1. Significance

Myofascial pain is a significant health problem affecting as much as 85% of the general population sometime in their lifetime while the estimated overall prevalence is ~46% [1, 2]. Myofascial pain syndrome is collection of the sensory, motor, and autonomic symptoms that include local and referred pain, decreased range of motion, and weakness. The health impact of myofascial pain can be quite severe as patients with the disorder not only suffer from decreased functional status associated with musculoskeletal pain and loss of function, but also suffer from impaired mood as well as decreased quality of life [3].

While myofascial pain syndrome is complex in its presentation, the onset and persistence of myofascial pain syndrome are known to be caused by myofascial trigger points [4]. In patients, myofascial trigger points present as focal areas in muscle that appear stiff and hypercontracted and are painful particularly when palpated. Despite the causal association of myofascial trigger points with the underlying physiology of myofascial pain syndrome, the mechanisms that induce the onset and maintenance of myofascial trigger points are unknown. Hence, a mechanistic understanding of myofascial trigger points is critical to developing treatments for myofascial pain syndrome. Critical insights will be gained by research addressing the following questions.

1. What initiates the formation of a myofascial trigger point?
2. What sustains a myofascial trigger point?
3. What causes a myofascial trigger point to be painful?
4. What will make the myofascial trigger point disappear?

## 2. Background

Myofascial pain syndrome arises from the muscle and is composed of symptoms from the sensory, motor, and autonomic systems [5]. Myofascial pain syndrome is caused by myofascial trigger points which are identified by palpation as discrete foci of hypercontracted areas within a muscle. Clinically, myofascial trigger points are defined as* active* or* latent*. An active myofascial trigger point is recognized as eliciting spontaneous pain as well as pain, referred pain, and motor or autonomic symptoms on palpation [6]. These include an impaired range of motion, muscle weakness, and loss of coordination. In contrast, latent myofascial trigger points upon palpation/compression cause pain, a local twitch response, and referred pain [7]. In fact they* may *display all the symptoms of an active trigger point to a lesser degree. For example latent trigger points may have associated autonomic symptoms with pain and their presence results in a limited range of motion, muscle fatigability, and muscle weakness as in the active presentation [8, 9]. This makes latent trigger points a significant concern also.

It is important to distinguish between myofascial pain and neuropathic pain. While myofascial pain originates at the muscle, neuropathic pain results from an injury to or malfunction of the peripheral or central nervous system [10]. There are myriad different pain syndromes and chronic pain disorder that fall into the category of neuropathic pain [11]. Myofascial pain, on the other hand, is thought to originate at the trigger point in the taut band of muscle.

In order to begin to gain mechanistic insights into the mechanisms of myofascial trigger points, it is helpful to consider aspects of skeletal muscle physiology.

*Excitation-Contraction Coupling*. Excitation-contraction coupling encompasses the processes involved in the neural activation of the muscle cell to the contraction and subsequent relaxation of the muscle fiber. Skeletal muscle, like cardiac muscle, is called striated muscle because it has a banded appearance within the single myocyte due to repeated pattern of contractile units termed as sarcomeres. Contraction of skeletal muscle is governed by motor neurons and graded by motor units, which are the collection of muscle fibers innervated by a single motor neuron (for review see [12]). At the neuromuscular junction (i.e., the interface between a motor neuron and a single muscle fiber), a motor neuron action potential initiates the release of acetylcholine from the presynaptic nerve terminals. Acetylcholine then diffuses across the synaptic cleft to activate nicotinic acetylcholine receptors in the postsynaptic membrane. Muscle contraction occurs when the summation of acetylcholine receptor activation reaches the threshold to trigger voltage dependent sodium channel activation in the sarcolemma outside the neuromuscular junction and subsequent action potential generation and depolarization of the muscle fiber. The clearance of acetylcholine from the synaptic cleft by acetylcholinesterase resets the process for subsequent activation.

The contraction of the muscle fiber is triggered by action potential transmission deep within the muscle fiber through sarcolemmal membrane invagination's termed as transverse tubules (t-tubules). Anatomic specialization within the t-tubule is seen at the triad where the t-tubule is flanked by the calcium storage organelle, the sarcoplasmic reticulum. The membrane depolarization accompanying the arrival of the action potential at the triadic space within the t-tubule activates voltage dependent L-type calcium channels in the transverse tubular membrane. Type I ryanodine receptors are located in the sarcoplasmic reticulum in close proximity to the L-type channels [13] and physical coupling between the type I ryanodine receptors and the L-type calcium channels (they either directly or through accessory proteins cause the ryanodine receptors to undergo a conformational change and open releasing calcium into the myoplasm) [14].

This transient rise in calcium binds to troponin on the actin thin filament, which relieves the inhibition on actin for binding by the contractile protein myosin. The calcium dependent interaction of actin and myosin occurs through the formation of strongly bound myosin crossbridges to actin. Force is then generated through ATP (adenosine triphosphate) dependent processes. Critical to this process is ATP hydrolysis that provides energy for the enzymatic activity of the myosin head which generates a single articulation of the myosin head and molecular movement of actin past myosin which shortens the sarcomere. The rebinding of a new ATP is then needed to relieve the strongly bound actin and myosin. In this process, muscle shortening occurs at a rate dependent on the speed of myosin's enzymatic activity and the resultant force and power output is the ensemble of the crossbridge cycle (i.e., attachment-myofilament sliding-detachment) of all myosin heads in each muscle. The crossbridge cycle is therefore calcium and ATP dependent and maintained as long as calcium and ATP remain high in the cytoplasm. In fact, a depletion of ATP while calcium is elevated results in the inability of crossbridge detachment and the formation of the “rigor bond” which leads to the stiffness seen postmortem.

Subsequent to the calcium release into the myoplasm, the sarcoendoplasmic reticulum ATPase (SERCA) works to sequester calcium back into the sarcoplasmic reticulum, again via ATP dependent enzymatic activity. Subsequent to a brief activation (~5 msec) by a single action potential, troponin, SERCA, and a host of other calcium binding proteins compete for calcium such that a brief force transient is realized (i.e., twitch). Grading force production at the single muscle fiber is then produced by delivering action potentials at higher frequencies (i.e., repetitive firing of the motor unit) resulting in pulses of calcium release that progressively increase myoplasmic calcium concentration.

*Energetics*. The molecule ATP is the critical energy source for muscle function. The metabolic processes that generate ATP use carbohydrates fatty acids and sometimes amino acids as their primary substrate (for a review see [15]). Carbohydrates are converted to glucose which enters the glycolysis pathway in the cell cytoplasm. The end product of this process is pyruvate which is converted to acetyl CoA by pyruvate dehydrogenase in the mitochondria. The *β*-oxidation of fatty acids results in formation of acetyl CoA. Acetyl CoA enters the tricarboxylic acid cycle (Krebs cycle) that takes place in the mitochondria [16]. Amino acids are converted to other tricarboxylic acid cycle intermediates and enter the cycle at different point. The tricarboxylic acid cycle produces the reducing equivalents NADH and FADH_2_ that enter the electron transport chain. The electron transport chain uses the energy contained in the reducing equivalents to pump protons out of the mitochondria producing an electrochemical gradient (pH gradient and membrane potential) that is used to make ATP [17]. The electron transport chain consumes oxygen during proton pumping resulting in the term oxidative phosphorylation to describe the entire process that transfers the energy in the reducing equivalents to ATP. Hence, the mitochondria in the myocytes provide the ATP needed for contraction. Skeletal muscles contain approximately 1–12% mitochondria by volume depending upon the particular muscle [18, 19]. Muscles with higher energy demand have higher mitochondrial content. For example, the diaphragm which is constantly active has 10–12% mitochondria by volume [18].

*Reactive Oxygen Species*. Striated muscle generates reactive oxygen species (ROS) which acts which modulates a host of biochemical processes including glucose uptake, gene expression, calcium signaling, and contractility through the targeted modification of specific protein residues. In striated muscle, contractile activity increases ROS signaling which leads to physiologic adaptation; however, in pathological conditions, ROS signaling is often in excess where it contributes to contractile dysfunction and myopathy.

Striated muscle generates superoxide as the primary ROS. Superoxide is generated by the addition of a single electron to ground state oxygen [20]. Superoxide is a highly reactive, unstable species that is rapidly converted by superoxide dismutase (SOD) to hydrogen peroxide (H_2_O_2_), a weaker but more stable oxidant. H_2_O_2_ is highly diffusible within and between cells, activates multiple signaling pathways, and is decomposed by either catalase or glutathione peroxidase to water and oxygen [21, 22].

The most well described source for superoxide production is the mitochondria where superoxide is produced within the electron transport chain (ETC) [23]. Recent work estimates that, at rest, the percentage of the ROS generated by electron flow through the ETC is low (<1%). During sustained vigorous contractile activity, however, where mitochondrial function increases >50-fold, the magnitude of superoxide release is modestly increased [24, 25], only a small amount (~2–4-fold) over resting levels (see [22] for review).

Two additional ROS sources are operant in muscle and yet are likely to be of significance only in disease or high stress conditions. The enzyme xanthine oxidase (XO) has been shown to produce superoxide in response to contractile activity in rodent muscle [20, 26]. Available evidence however supports either a vascular source of XO generated superoxide due to contractile shear stress or an increase in XO activity secondary to anaerobic metabolism that increases the availability of XO substrates [26–28]. Superoxide is also produced by phospholipase A2 (PLA2) dependent processes [29] with a Ca^2+^-independent PLA2 contributing to ROS production under resting conditions [30], whereas a Ca^2+^-dependent PLA2 may contribute to ROS production during contraction when cytosolic [Ca^2+^] is elevated [31–33]. While XO or PLA2 sources of ROS are unlikely to play a role in the sustained ROS production at rest or during dynamic contractions, each supports a mechanism to increase superoxide following exhaustive/fatiguing contractions or sustained contractures where anaerobic metabolism predominates.

Recent work in striated muscle by Ward and coworkers and others has implicated nicotinamide adenine dinucleotide phosphate oxidase 2 (NADPH oxidase; NOX) as the major source of superoxide ROS during repetitive contraction [24, 25]. NOX is a multimeric enzymatic complex that generates superoxide by transferring electrons from NADPH to oxygen. Several NOX isoforms are expressed in striated muscle and located within the sarcoplasmic reticulum, the sarcolemma, and the transverse tubules [34, 35]. Striated muscle cells express NOX2 and 4 which each bind p22^phox^, a small subunit essential for enzyme activity [34]. NOX4 is constitutively active and does not require association with regulatory subunits, with regulation thought to occur mainly by changes in expression level. Therefore NOX4 likely contributes to the basal rate of ROS production in the myocyte. In contrast, NOX2 (also known as gp91^phox^) is activated by specific agonists (e.g. G-protein coupled receptor agonists such as angiotensin II, growth factors, and cytokines) and mechanical/contractile stress, which induce the association of regulatory subunits (p47^phox^, p67^phox^, p40^phox^, and Rac1) and activation of the enzyme [35, 36].

The mechanosensitivity of NOX2 has recently garnered much attention. The production of reactive oxygen species by NADPH oxidase is regulated by the small Rho like GTPase protein Rac1 [37, 38]. The Rac1 protein activity is regulated by microtubules [39] and the actin cytoskeleton [39]. During mechanical stress such as the stretching/twisting of airway smooth muscle cells, the cytoskeleton deforms and activates Rac1 [40]. This response is disrupted if myosin II tension is blocked with blebbistatin, f-actin is disrupted with cytochalasin D, or the microtubules are disrupted with colchicine [40]. This mechanism is also present in skeletal muscle where mechanical stretching induces ROS production by NOX2. This mechanoactivation of NOX2 dependent ROS production has been recently shown to be critical to the pathogenic calcium and ROS signaling in Duchenne muscular dystrophy. Relevant hypothesis on mechanoactivated NOX2 dependent ROS production is discussed below.

## 3. Myofascial Trigger Point Mechanisms

In all cases, myofascial trigger points are associated with areas in muscle that have stiff, tender nodules under palpation. It is believed that this stiffness might arise from hypercontracture of the sarcomere in this area [41, 42]. Histological examination of muscle biopsies from myofascial trigger points reveals structural evidence of muscle hypercontracture consistent with sustained sarcoplasmic reticulum calcium release due to intense neural activation and action potential generation [42]. This is supported by the work identifying trigger points exhibiting spontaneous electrical activity suggesting aberrant action potential generation [43]. Further pathological findings associated with sustained hypercontraction/activity (e.g., sarcomere shortening, protein degradation, and myofiber and mitochondrial swelling) are consistent with metabolic stress and ATP depletion.

**Figure 1:**
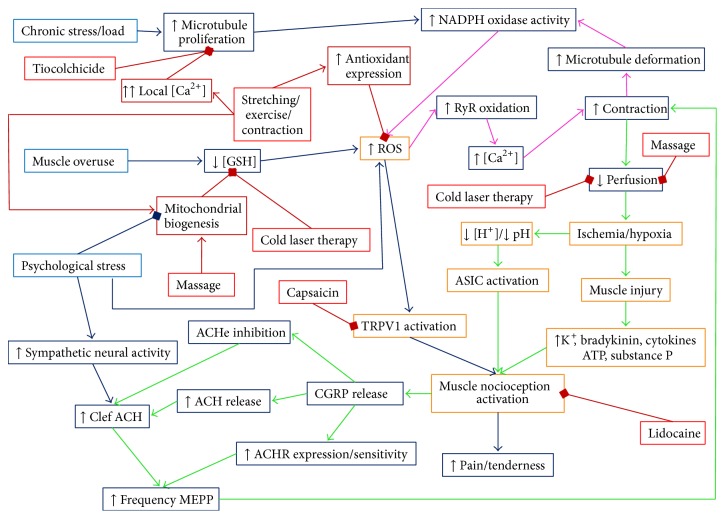
Hypothesized signaling pathways of myofascial trigger points and therapies. A schematic diagram of the mechanism of myofascial trigger points and treatment options. The initiating mechanisms of myofascial trigger points are shown in blue with the downstream events in dark blue. The treatment for myofascial pain is shown in red with the pathways of their action shown in dark red. The arrows indicate how one feature causes another. The square at the ends of lines indicates inhibition of the end feature by the preceding feature. The myofascial trigger point is initiated by a combination of chronic load on the muscle which caused microtubule proliferation which increases ROS production and a decreased ability to remove ROS. The increase of ROS increases ryanodine receptors open probability, hence increasing calcium which results in contraction and deformation of the microtubule network resulting in more ROS production. This is the key positive feedback loop. Psychological stress can contribute to this as it reduces mitochondrial content and increase ROS production in cells. The contraction restricts blood flow resulting in local ischemia/hypoxia that results in muscle damage and the inflammatory response. Pain is caused by the activation of nociceptors by a decreased pH (ASIC channels), increased ROS (TRPV1 channels), and substance P. When depolarized, nociceptive neurons release CGRP which increases the amount of and response to acetylcholine in the neuromuscular junction, which can cause additional contraction. This provided a second positive feedback loop. Treatments such as lidocaine and capsaicin block nociception. Other treatments such as massage and cold laser therapy might increase circulation or reduce oxidative stress. Furthermore, treatments that induce stretching/contraction such as needling, electrical stimulation, stretching, and exercise increase calcium local to high enough concentrations (>10 *μ*M) transiently that induce microtubule depolymerization. Finally, the application of topical or injected thiocolchicine has been shown to reduce myofascial pain. Abbreviations: GSH (glutathione), ROS (reactive oxygen species), NADPH (nicotinamide adenine dinucleotide phosphate), RyR (ryanodine receptors), [Ca^2+^] (calcium concentration), [H^+^] (proton concentration), ATP (adenosine triphosphate), TRPV1 (transient receptor potential cation channel subfamily V member 1 or capsaicin receptor), ASIC (acid-sensing ion channel), CGRP (calcitonin gene related peptide), ACH (acetylcholine), ACHe (acetylcholinesterase), and ACHR (acetylcholine receptor).

Sustained contractile activity leading to increased metabolic stress and reduced blood flow is likely the foci for secondary changes that contribute to the persistence of the myofascial trigger point. In addition the sustained contractile activity, metabolic alterations, and cell stress trigger the increased release of myokines, inflammatory cytokines, and neurotransmitters that also undoubtedly contribute to these myofascial trigger points and myofascial pain syndrome.  Figure 1 shows the mechanisms of initiation and maintenance myofascial trigger points, how they cause pain, and how current therapies work. These pathways will be explained in the following sections.

### 3.1. Initiation of Myofascial Trigger Points

A clear mechanistic description for the initiation of a myofascial trigger point does not currently exist. Trigger points are thought to occur as a result of muscle overuse or muscle trauma or psychological stress [5]. Examples include trigger points arising secondary to muscle overload in worksite tasks or activities of daily living such as lifting heavy objects or sustained repetitive activities. In these cases, poor ergonomics, improper postural positioning, deconditioned muscle, and fatigue have been associated with the development myofascial trigger point. While muscle conditioning has been shown to reduce the incidence, the occurrence of myofascial trigger points in elite athletes suggests a threshold above which inciting events may initiate a myofascial trigger point [44]. Psychological stress may complement these mechanisms in the development of myofascial trigger point [45, 46].

Another important consideration is that, in some healthy individuals and athletes, muscle fatigue or trauma does not always result in myofascial trigger points. Instead they can result in stiffness, soreness, and pain that usually resolve themselves after a few days. This also supports a threshold for occurrence and/or possibly a cofactor that promotes the initiation of a myofascial trigger point. Here, clues to the mechanistic events that initiate myofascial trigger points may be gained from our knowledge that comorbid conditions such as aging, disease, and stress increase the incidence of myofascial trigger points. For example, myofascial trigger points are thought to underlie the spontaneous pain patter in individuals suffering from fibromyalgia [47, 48]. Trigger points also have been observed with an increased frequency in patients suffering from reflex sympathetic dystrophy which is thought to arise from the unique emotional and psychological condition of these patients and psychological stress in other patients [45, 46, 49, 50]. Additionally, myofascial trigger points may arise secondary to iatrogenic causes as certain cancer therapeutic regimens (i.e., taxanes and pacilitaxil) induce myofascial pain [51, 52].

Myofascial trigger points are more common under conditions of psychological stress [46]. In fact, myofascial trigger points display increased myogenic activity, while the adjacent muscle remained silent under psychological stress [45]. Psychological stress results in an increase of certain hormones and increase of sympathetic neural stimulation. It is believed that the increase in hormones and sympathetic stimulation during this condition leads to increase in release of acetylcholine at the neuromuscular junction contributing to the contraction of the motor units involved in a trigger point [53]. This and other mechanisms that initiate a myofascial trigger point must feed into the mechanisms for their persistence described in the next section.

### 3.2. Mechanisms for Persistence of Myofascial Trigger Points

The persistence of myofascial trigger points requires a self-sustaining positive feed-forward process. Simons presented the integrated hypothesis for myofascial trigger points to offer an explanation [4]. The integrated hypothesis is a six-link chain that starts with step (1): the abnormal release of acetylcholine. This triggers step (2): increased muscle fiber tension which is seen as the taut band found in a myofascial trigger point. The taut band is thought to constrict blood flow that leads to step (3): local hypoxia. The reduced oxygen disrupts mitochondrial energy metabolism reducing ATP and leads to step (4): tissue distress and step (5): the release of sensitizing substances. These sensitizing substances lead to pain by activation of nociceptors (pain receptors) and also lead to step (6): autonomic modulation that then potentiates step (1): abnormal acetylcholine release.

More recently this hypothesis has been expanded by Gerwin and coworkers [53]. It suggests more specific details of the feedback loop. For example, sympathetic nervous system activity augments acetylcholine release as well as the local hypoperfusion caused by the muscle contraction. The resulting ischemia/hypoxia leads to acidification (decreased pH). Experiments have shown that injections of acidic saline of pH 4 can cause muscle pain through activation of muscle pain receptors called acid-sensing ion channels (ASIC3) [54, 55]. While this low pH is much lower than that seen during ischemia, a smaller physiological decrease in pH has been shown to activate ASIC3 channels [56]. The prolonged ischemia/hypoxia also leads to muscle injury resulting in the release of potassium, bradykinins, cytokines, ATP, and substance P which might stimulate nociceptors in the muscle [53, 57]. The end result is the tenderness and pain observed with myofascial trigger points accompanied by calcitonin gene-related peptide (CGRP). Depolarization of nociceptive neurons causes the release of CGRP [58]. CGRP inhibits acetylcholine esterase and upregulates the amount of acetylcholine receptors and release of acetylcholine. This nonquantal spontaneous acetylcholine release at the motor end plate as a result of CGRP is termed as acetylcholine leakage [59]. This differs from the other modes of acetylcholine release such as simulation induced multiquantal release resulting in an end plate potential (EPP) and spontaneous quantal releases resulting in a miniature end plate potential (MEPP) [59]. The theory also postulates CGRP release from nerve terminals with the same targets. Furthermore, a decrease in pH can also cause an increase in acetylcholine release [60]. The result is increased acetylcholine in the nerve terminal, synaptic cleft, and increased motor endplate potentials resulting in more contraction [61, 62]. The model also suggests that psychological stress also increases acetylcholine release into the neuromuscular junction.

These expanded hypotheses presented in the previous paragraph are based upon experimental and clinical evidence and understanding when possible [53, 63]. Using microdialysis samples of the chemical milieu of the muscle can be obtained. In microdialysis, a hollow needle filled with an absorbing gel and with a semipermeable membrane at its tip is inserted into the myofascial trigger point. Another is inserted into adjacent normal muscle for comparison. Ions, signaling molecules, and proteins diffuse into the gel that does not leave the needle. These are assayed upon removal of the needle. While this method allows access to the muscle interior, it cannot differentiate between the intracellular and extracellular spaces. Such experiments, hence, can give an idea of the chemical species in the muscle tissue but not specifically describe the intracellular conditions of the skeletal muscle cell.

The positive feedback loop in the above mechanism requires that there be sustained stimulation of the muscle motor unit due to increased acetylcholine release and decreased acetylcholinesterase activity. However, it appears that acetylcholine release might not be required for sustaining trigger points. In studies comparing the efficacy of motor nerve block using lidocaine injection compared to intramuscular stimulation using dry needling, the group receiving the intramuscular stimulation showed more than 40% greater improvement than did the lidocaine injection group [64]. Furthermore, lidocaine shows a dose-dependent decrease in miniature end plate potential, acetylcholine release, and acetylcholine sensitivity [65, 66]. Therefore, there might be another mechanism that provides the positive feedback that sustains a myofascial trigger point. However, if the spontaneous nonquantal acetylcholine release is a direct result of CGRP, it might be too small to measure and, since it does not involve nerve depolarization, would be unaffected by lidocaine.

### 3.3. Biophysical Perspectives of Current Therapies for Myofascial Trigger Points

There are several therapies currently used to treat myofascial trigger points including massage, stretching, dry needling/injections, electrical stimulation, cold laser treatment, and ultrasound. There are several massage treatments that relax myofascial trigger points such as passive rhythmic release, active rhythmic release, and trigger point pressure release [4]. This paper is not intended to be a comprehensive discussion of drugs that are used to treat trigger points. What seems to be common to the therapies is that they alleviate muscle stiffness and pain and may be combined with therapies that stretch and improve metabolism at the hypercontracted trigger point region.

#### 3.3.1. Massage

It is widely believed that massage increases blood flow. For example, in one study massage of the lower left extremity in young females increase blood flow in the tibial artery as measured by Doppler ultrasound [67]. If this occurred on the local level in muscle, this would in principle break the cycle in the above theories. However, this finding does not necessarily affect the microcirculation which is thought to be constricted in a myofascial trigger point. Furthermore, another study of the benefits of massage after exercise focused more on the muscle and found that there was no significant increase in circulation after massage [68]. Instead, massage activated the mechanotransduction signaling pathways FAK and ERK (focal adhesion kinase and extracellular signal-regulated kinase, resp.), decreased inflammatory cytokines, and increased mitochondrial biogenesis [68]. Mitochondrial biogenesis by increasing the amount of mitochondria would improve energy metabolism in the muscle. The NADPH oxidase/Rac1 increases the autophosphorylation of FAK. FAK is a scaffold for EGF-mediated signaling including activation of ERK. Furthermore, inhibition of NADPH oxidase/Rac1 increases focal adhesions [69]. ERK activation potentiates FAK-stimulated removal of focal adhesions [70]. Focal adhesions have been implicated as a component of stiffness in aortic smooth muscle cells [71]. Therefore, one way massage may reduce muscle stiffness is by the reduction of focal adhesions through activation of the FAK pathway.

#### 3.3.2. Stretching

Stretching of muscle involves a series of stretching exercises of the muscle where pain is experienced [4]. The reason that this works is not clearly understood. Anecdotally, it is thought to increase blood flow to muscle; however, it is unclear if this is supported by experimental studies. In fact, stretching of muscle transiently decreases blood flow proportionally to the amount of stretching [72–74]. This is due in part to the longitudinal stretching of the blood vessels running in the direction of the muscle fibers [73, 75]. It is also due to the compression of the blood vessels by the increase in intramuscular pressure [76, 77]. On the other hand, muscle stretching training may actually increase circulation as shown in ballet trained individuals [78]. This might explain the observed long-term benefit. There also might be other mechanisms involved. Recent experiments have shown that stretching skeletal myocytes activates NADPH oxidase [79]. This occurs through the microtubules serving as mechanotransducers that interact with Rac1 that activates NADPH oxidase. This results in the production of reactive oxygen species that increase the open probability of the ryanodine receptors with no increase in channel conductance through the oxidation of thiol groups on the protein [80–83]. In muscular dystrophic cells this response is greater [79] due to the increase in microtubule density in these myocytes [84]. It should be noted that reactive oxygen species from any source displays an increase in ryanodine receptor open probability. For example, reactive oxygen species from hypoxanthine/xanthine oxidase has also been shown to increase ryanodine receptor open probability [85]. In addition, this presents the possibility that stretching of muscle, similar to massage, activates the FAK and ERK pathways.

#### 3.3.3. Dry Needling/Injections

The insertion of a needle (acupuncture) can release a myofascial trigger point if the insertion of a needle into the trigger point elicits a local twitch. This local twitch involves a transient increase in activity in the muscle band containing the trigger point. Furthermore, it is considered to be a spinal reflex since spinal cord transection between the brain and the level of the trigger point does not affect the response [86]. A local twitch at the site is thought to stretch the muscle fibers at that location [87]. The relaxation of the muscle after twitch is thought to relieve constriction of the capillaries which restores the microcirculation. This reoxygenates the muscle at the site of the trigger point breaking the positive feedback. Recent studies indicate that dry needling increases blood flow and oxygenation to the muscle band containing the trigger point and not the rest of the muscle [88].

#### 3.3.4. Electrical Stimulation

Electrical stimulation places electrode across the muscle affected by a trigger point and rapidly causes contractions by depolarizing the muscle. The goal of this therapy is to increase the size and frequency of the twitches that could have been elicited by needling [87]. Once again this might work through the mechanism of stretching. In fact, muscle stimulation was shown to be more effective in treating trigger points than application of lidocaine [64]. It has been proposed that working the muscle in this fashion could be more like exercising the muscle. For example, in one study voluntary and electrically evoked isometric contractions showed similar oxygen demand at maximum intensity although the torque generated by voluntary isometric contraction was 40% of the torque generated by electrically evoked isometric contraction [89].

#### 3.3.5. Cold Laser Therapy

Cold laser therapy also known as low level light therapy exposes the myofascial trigger point to near infrared light. It has been shown to work clinically reducing pain and rigidity and increasing mobility [90, 91]. This study suggests that the increased motion leads to an increase in microcirculation. Others claim that cold laser therapy is thought to energize the mitochondria. In mouse embryonic fibroblasts low level light therapy increased intracellular ATP and reactive oxygen species levels [92]. The source of the increased reactive oxygen species is likely mitochondria [93]. Cold laser therapy has been shown to reduce oxidative stress in skeletal muscle [94].

#### 3.3.6. Ultrasound Therapy

Ultrasound is often used to treat myofascial pain and trigger points. However, the benefits are unclear. While exercise and massage seem to reduce pain and the number and size of myofascial trigger points, conventional ultrasound did not result in pain reduction [95]. On the other hand, high powered ultrasound before stretching increases mobility more than conventional ultrasound [96]. This might be due to the heating effect of ultrasound. For example, thermal ultrasound has been shown to reduce stiffness of myofascial trigger points [97].

## 4. New Mechanistic Theory for Myofascial Trigger Points

In light of the discussion above, it is clear that the current theory for the mechanisms behind myofascial pain is not sufficient to fully explain the syndrome. As the myofascial trigger point appears central to the onset and persistence of myofascial pain syndrome, we have focused attention on the muscle fiber level in an attempt to reveal new mechanistic insight. Here we present mechanistic findings in muscle that demonstrate how mechanical stress acts to trigger excess calcium release in muscle via a novel mechanotransduction pathway. With this new pathway as a foundation, we put forth a novel mechanistic hypothesis for the initiation and persistence of myofascial trigger points that extends the current theories discussed above.

### 4.1. What Initiates and Sustains a Myofascial Trigger Point?

The essence of this question is what positive feedback mechanisms exist that can sustain a myofascial trigger point once initiated. Based on the models and mechanisms discussed above, the local and persistent hypercontracture of the muscle appears to be critical to the myofascial trigger point. At the cellular level, a persistent neural activation may act to initiate a local and sustained contraction; however, fatigue of the muscle would ensue much as in a highly trained athlete with high motivation that is eventually unable to sustain muscle activity. Rather, the local contracture of the muscle must occur secondary to the normal neuromuscular activation and arise due to regenerative feed-forward processes within the muscle cell. At the most basic level, this situation would necessitate a mechanism that permitted regenerative calcium release within the myofibers that escaped from the normal inhibitory processes that govern central and peripheral muscle fatigue. It would be most practical for this feed-forward mechanism to take advantage of any aberrant activity (contraction dependent mechanical stress, calcium release, and altered metabolic signaling) as an initiation trigger and as a mechanism to sustain its activity.

X-ROS signaling is anewly characterized mechanoactivated ROS-dependent signaling cascade in cardiac and skeletal muscle. In X-ROS signaling mechanical deformation of the microtubule network acts as a mechanotransduction element to activate the NADPH oxidase (NOX2) which produces ROS. The ROS oxidizes RyRs and increases their open probability resulting in increases in Ca^2+^ release from the sarcoplasmic reticulum. The Ca^2+^ mobilization resulting from mechanical stretch through this pathway is X-ROS signaling. In heart, X-ROS acts locally to affect the sarcoplasmic reticulum (SR) Ca^2+^ release channels (ryanodine receptors, ryanodine receptors) and “tunes” excitation-contraction coupling Ca^2+^ signaling during physiological behavior but can promote Ca^2+^-dependent arrhythmias during pathology with X-ROS in excess [79]. In skeletal muscle, X-ROS sensitizes Ca^2+^-permeable sarcolemmal “transient receptor potential” (TRP) channels, a pathway critical for sustaining SR load during repetitive contractions. When in excess, X-ROS in skeletal muscle is maladaptive as shown in diseases such as Duchenne muscular dystrophy (DMD) and dysferlinopathy which both have altered calcium signaling as major mechanistic underpinnings. Importantly, work in DMD by Khairallah et al. suggests that the development of X-ROS (i.e., enhancement in the expression of microtubule protein and NOX2 and its resultant increase in mechanoactivated ROS) is a secondary process that is temporally associated with the severity of the disease and not a primary cause of the disease [84, 98]. In that regard the enhancement in X-ROS was a disease modifier that increased the severity of the disease by lowering the threshold for calcium release in the muscle.

The above mechanism purports that excessive contraction dependent stress acts through the microtubule cytoskeletal elements to activate NADPH oxidase to produce ROS. The subsequent ROS sensitization of ryanodine receptors and sarcolemmal calcium influx channels increases myoplasmic calcium concentration and contraction leading to more stretch. Based on this model, one reason why the occurrence of myofascial trigger points may be less common in healthy individuals is due to the absence of the feed-forward trigger, excess microtubules, or NOX2 that serves to generate X-ROS. This hypothesis then proposes that the threshold for developing myofascial pain and myofascial trigger points is lower with the trigger present as a critical amount of X-ROS activity that would serve to lower the threshold for calcium release activation such that spontaneous or regenerative calcium release generation promoted can initiate the contractures which underscore the myofascial trigger point.

Our hypothesis that the microtubule cytoskeleton and X-ROS may play a role in the mechanism of myofascial trigger point came from work that suggests that microtubule proliferation have been associated with either myofascial trigger points or myofascial pain or both. Taxane based chemotherapy, for example, pacilitaxel, is a common therapy for cancer as by increasing microtubules and it blocks the organization of the centrosome and kinetochore thereby inhibiting mitosis [99]. A significant fraction of patients under cancer therapy with paclitaxel show an increase in myofascial pain [100]. It is therefore tempting to speculate that a drug-induced increase in the microtubule network in muscle acted to increase contraction-induced X-ROS and lowered the threshold for the development of myofascial trigger point and myofascial pain. We are actively investigating this new concept.

Myofascial trigger points are thought to occur by muscle injury and muscle overuse [53]. In heart muscle the increased mechanical stretch and increased mechanical load underpin heart failure and result in increases in microtubule density and polymerization [101, 102]. Furthermore, in cardiac muscle prolonged stretching increases mitochondrial biogenesis through the focal adhesion kinase (FAK) signaling pathway [103]. Blocking this pathway using RNAi attenuated the increase in mitochondrial biogenesis. If chronic functional overload of skeletal muscle also resulted in microtubule dependent effects, this could offer an explanation for muscle imbalances leading to functional overload of muscle such as the trapezius which is prone to myofascial trigger points and foci for myofascial pain in the upper back. As the effect of functional skeletal muscle overloading on microtubule proliferation has not yet been directly investigated, we are seeking new models to address this question.

One intriguing possibility arises. Recently, noninvasive imaging studies using Doppler ultrasound or vibration elastography have been shown effective in detecting myofascial trigger points. Areas associated with myofascial trigger points are hyperechoic under ultrasound imaging having lower vibration amplitude and entropy, respectively, than that of normal muscle [104]. This finding is consistent with increased tissue density [105] which is indicative of a contracture or proliferation of a protein. In fact, in cardiac muscle, increasing microtubule polymerization by application of taxol increases tissue viscosity. Depolymerization of microtubules by colchicine had the opposite effect [106]. As microtubule density has been shown to correlate with cell viscosity/stiffness [107], we hypothesize that the hyperechoic regions in the ultrasound of myofascial trigger points [105] are reflective of an increase microtubule density. Our new focus is marrying this imaging technology to new animal's models in development to mechanistically address this hypothesis.

In addition to the increase of ROS production through the X-ROS mechanism, a reduced ability of the muscle cell to remove ROS most likely also plays a role in the mechanism. In normal stretching of the muscle, reactive oxygen species are removed by the normal homeostatic mechanisms. Superoxide is reduced to hydrogen peroxide by superoxide dismutase. The hydrogen peroxide is removed by catalase or glutathione oxidase and converted to water. The glutathione that is oxidized to glutathione disulfide is converted back to glutathione by glutathione reductase consuming NADPH in the process. The regeneration of NADPH uses the nicotinamide nucleotide transhydrogenase which requires a proton gradient and membrane potential across the mitochondrial inner membrane. The local ischemia that results from the formation of a myofascial trigger point will result in a decrease in mitochondrial membrane potential and increase in extramitochondrial proton concentration (decreased pH) [108, 109]. This reduces the ability of the muscle to remove reactive oxygen species and should contribute to the maintenance of high reactive oxygen species level that leads to sustained myofascial trigger points.

As mentioned previously, muscle-damaging exercise and psychological stress play a role in the initiation of myofascial trigger points. This might be due to the reduced ability of the muscle to remove ROS under these conditions. Experimental studies have shown that repeated muscle-damaging exercises results in muscle oxidative stress which includes decreased levels of glutathione and increased levels of its oxidized form, glutathione disulfide [110]. Psychological stress increases mitochondrial biogenesis in the short term [111, 112]. On the other hand, sustained exposure to the hormones produced during psychological stress decreases mitochondrial biogenesis [112, 113]. Furthermore, this prolonged stress increases mitochondrial ROS production [111, 112, 114]. This increased ROS production might further deplete antioxidant defense systems, increasing overall cellular reactive oxygen species levels.

**Figure 2:**
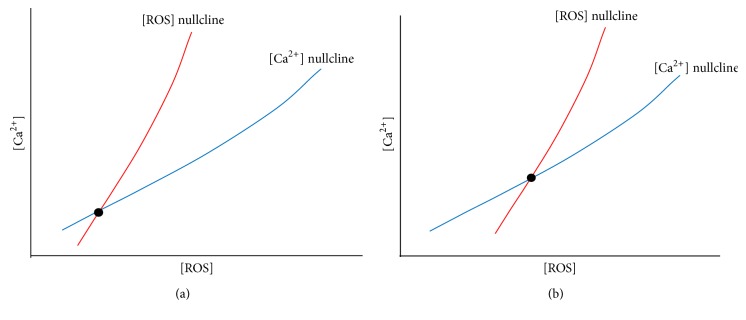
Hypothesized mechanism yielding active and latent trigger points. At the site of the myofascial trigger point, there is restriction of blood flow increasing the proton concentration ([H^+^]) locally. There is also an increase of local [ROS]. The plot shows the normalized concentrations of these substances as a function of the distance from the trigger point center. As the distance from the trigger point center increases, the levels of [ROS] (blue) decrease more gradually than the levels of [H^+^] (red). If the nociceptors are located close to the center of the trigger point, they are active as the levels of [ROS] and [H^+^] protons are high enough to activate the ASIC and TRPV1 channels. If these neurons/receptors are farther away the, trigger point would be latent. Upon palpation the [ROS] increases due to mechanical deformation of the microtubule network and activation of NADPH oxidase and the [ROS] increases (green) so that the nociceptors see sufficient ROS to be activated.

In summary, we hypothesize that the occurrence of X-ROS is a disease modifier for the development of myofascial trigger point. We propose that the molecular underpinning of the myofascial trigger point involves an increased microtubule density which leads to an increase in NOX2-dependent reactive oxygen species production which in turn lowers the threshold for calcium release and entry in muscle. In the trigger point region there will be increased ROS levels as well as increased calcium levels. In fact, it has been hypothesized that calcium is elevated in myofascial trigger points [115]. Hence, the trigger point is a new steady-state condition of the muscle with altered physiology. This can be demonstrated in a phase plan diagram showing the nullclines for calcium and ROS (Figure 2). A nullcline is the curve where the derivatives for the differential equations for calcium and ROS are set to zero. Their intersection is the steady state of the system. Under normal conditions the calcium and ROS levels are low at steady state (Figure 2(a)). When ROS production increases and its removal decreases, the ROS nullcline shifts to the right (high ROS levels) and it intersects the calcium nullcline so that the steady state results in higher ROS and calcium levels (Figure 2(b)). Hence, with myofascial trigger points the physiology of the system has been shifted so that there is a new steady state. To return the system back to normal, the physiological changes need to be reversed.

### 4.2. What Causes a Myofascial Trigger Point to Be Painful?

Myofascial trigger points yield pain upon palpation. If they are only painful upon palpation, they are called latent. If they are painful without manipulation they are considered to be active [116]. Given current information, there are two possible and perhaps complementary mechanisms behind the pain experienced as a result of myofascial trigger points. These two mechanisms involve the nociceptors ASIC3 (acid-sensing ion channel) and TRPV1 (transient receptor potential) channel in pain sensing neurons. These mechanisms are described below.

The local sustained contraction in a myofascial trigger point can result in restriction of local circulation which can cause the local ischemia/hypoxia and the observed changes caused by it such as increased acid accumulation resulting in a decrease in pH. In experiments in rabbit gastrocnemius muscle, the intracellular pH dropped from 7.0 to 6.6 during 4 hours of ischemia [108]. In the extracellular fluid the pH changes from 7.3 to 6.36 during the same time period due to the accumulation of lactic acid. There are four ASIC channels: ASIC1, ASIC2, ASIC3, and ASIC4. However, only ASIC3 seems to be involved in inflammatory pain [117, 118]. This pH drop is enough to fully activate the nociceptive ASIC3 channels in nearby neurons [56]. The pH changes seemed to level off due to a drop in membrane potential from −90 mV to −60 mV which resulted in increased proton extrusion.

As noted above, reactive oxygen species are produced in large amounts as a result of the mechanism of myofascial trigger points. There are several TRP (transient receptor potential) channels that respond to reactive oxygen species including TRPM2, TRPM7, TRPC5, TRPV1, and TRPA1 [119]. The TRP channels involved in pain are TRPV1–V4, TRPA1, and TRPM8 [120]. These two sets intersect at the TRPV1 channel making it a likely contributor. In fact, recent experiments have shown that reactive oxygen species can activate nociceptors in pain sensing neurons and enhance inflammatory pain [119]. Furthermore, the TRPV1 channel is also known as the capsaicin receptor. Capsaicin applied topically has been shown to reduce myofascial pain [121, 122].

**Figure 3:**
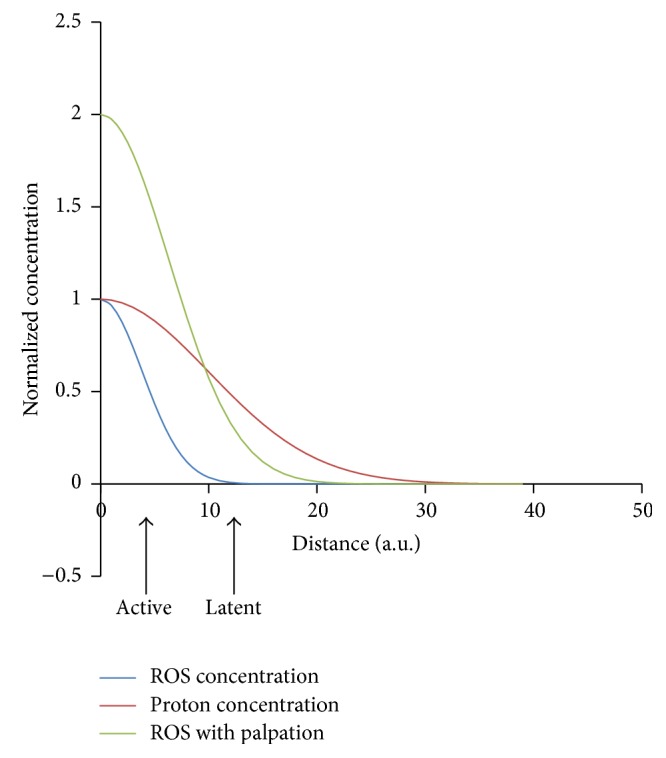
Hypothesis of how changes to the affected region cause a sustained perturbation yielding a trigged point. Conceptual phase plane diagrams for intracellular [Ca^2+^] and [ROS]. (a) The [ROS] nullcline (red) intersects the [Ca^2+^] nullcline (blue) at the steady state value for resting [Ca^2+^] and [ROS] (black dot). (b) With the changes that occur with microtubule proliferation and reduction in the ability of the myocyte to remove ROS, the [ROS] nullcline shifts to the right higher [ROS] for a given level of [Ca^2+^] resulting in a higher steady-state [ROS] and [Ca^2+^].

Considering that the two nociceptors above most likely are involved in the sensation of pain in the environment of trigger points, the question arises—why are some trigger points latent and some active? We hypothesize that pain is likely felt with manipulation of trigger points as the cytoskeleton is being stretched and reactive oxygen species production increased activation of more nociceptors. Some trigger points might be active because the local extracellular reactive oxygen species concentration in the location of the TRPV1 receptors is high enough for their activation. This requires that these receptors be close enough to the trigger point (Figure 3). Shown are three curves for the ROS level (blue), proton concentration (red), and ROS level upon palpation (green). The active trigger points are likely located closer to pain receptor channels so that the receptors see high levels of protons and ROS. Hence both ASIC3 and TRPV1 channels are activated. Latent channels are farther away so that they see lower levels of ROS (which dissipates faster than the pH gradient as ROS are unstable and larger molecules). Experimental studies have indicated that pressing on a cell activates stretch-activated channels causing increases in myoplasmic calcium [123]. This suggests that palpation might also trigger stretch-activated processes such as X-ROS signaling. Therefore, when palpated, the ROS levels might rise resulting in activation of the TRPV1 channels causing pain.

There are also other receptor channels that are located in the pain sensing neuron that are involved in the sensation of pain in myofascial pain syndrome [124]. The bradykinin receptors (B1 and B2) are involved in inflammatory pain. Serotonin receptors likely do not depolarize the neuron due to the low levels of serotonin in tissues but instead sensitize the neurons to activation by other factors. Prostaglandin (particularly E2) also sensitizes the pain sensing neuron. The P2X3 receptors are activated by ATP and its derivatives which can be released as a result of muscle injury. Glutamate receptors might also be involved as glutamate injections have been shown to lower the pressure pain threshold in patients [125].

In summary, it appears that myofascial pain is likely due to a combined activation of several ligand gated ion channels in the pain sensing neuron. For example, ASIC3 and TRPV1 open as a result of increased acidity and reactive oxygen species, respectively. Any treatment for pain should address these mechanisms.

### 4.3. What Will Make the Myofascial Trigger Point Disappear?

This is important for understanding what therapies can be used to treat myofascial trigger points. We can begin by explaining why current therapies work or do not work. The current therapies for myofascial trigger points are described above. These therapies include massage, stretching, dry needling/injection, electrical stimulation, cold laser therapy, and ultrasound. The underlying process with the treatment of trigger points is to temporarily release the trigger points to reduce pain and increase muscle mobility. This is often accomplished by massage, heat (direct or through ultrasound), and needling and injection for persistent trigger points. This is followed by stretching and simulation which essentially exercises the muscle. We hypothesize that the exercise of the muscle most likely starts to remodel the cytoskeleton, including the microtubular network, toward a more normal phenotype. There is also improvement in metabolism possibly by the increased blood flow and increase in mitochondrial content.

Injections of substances such as lidocaine and capsaicin block the activation of the nociceptive neurons. Capsaicin blocks activation of the TRPV1 channels while lidocaine blocks neuron depolarization (Figure 1). These however do not seem to disrupt the positive-feedback loop that sustains the myofascial trigger point. As mentioned previously, this could be because lidocaine's block of the sodium channel does not impair the CGRP-mediated acetylcholine leak into the neuromuscular junction that might contribute to the persistence of the trigger point.

The long-term effects might be from other aspects of the treatments that reduce oxidative stress. For example, some reports indicated that massage increases circulation. If this can restore circulation to an ischemia area, the mitochondria might recover and the removal of reactive oxygen species can be more effective. Massage might also increase mitochondrial biogenesis which can also help to relieve oxidative stress. Cold laser therapy also seems to reduce oxidative stress.

Another commonality between treatments is that there seems to be some sort of stretching involved and perturbation of the NADPH oxidase system. Massage and stretching directly stretch the muscle and can activate NADPH oxidase. Needling requires that a muscle twitch be elicited and electrical stimulation builds upon this by creating repeated strong twitches. This can lead to stretching as well.

Exercise has also been shown to help alleviate myofascial trigger points. During exercise there are transient elevations of calcium that are likely higher at peak than those seen in the muscle during a myofascial trigger point. Calcium-dependent regulator protein inhibited polymerization of microtubules at physiological calcium concentrations [126, 127]. This value is in the range of 10–100 *μ*M which is seen near the calcium release sites in muscle. At the site of calcium release during a calcium spark, calcium is predicted to be as high as 150 *μ*M by computational modeling [128, 129]. Therefore, exercise might reduce microtubule proliferation and hence ROS production from the NADPH oxidase. Furthermore, exercise also acts to increase the ability of the muscle to remove ROS. Moderate exercise upregulates antioxidant enzymes [130, 131].

Exercise also leads to mitochondrial biogenesis [132]. A final benefit of exercise is that it increases mitochondrial function as measured respiration and content as measured by citrate synthase activity [133].

If the hypothesis that increased microtubule polymerization results in a pathologic increase in reactive oxygen species production through the NAPDH oxidase complex, then treatments that reduce microtubule polymerization should show benefit for treating myofascial trigger points. In fact, administration of colchicine and related compounds seems to reduce myofascial pain. The application of topical thiocolchicoside reduced pain in patients with acute cervical myofascial pain to alleviate back pain in patients [134]. Oral colchicine, however, did not seem to alleviate back pain in a study by Schnebel and Simmons [135]. Another study by Simmons and coworkers indicated that intravenous colchicine administration reduced lower back pain in patients [136]. These findings seem to support the idea that microtubules play a major role in myofascial pain syndrome.

## 5. Conclusions

Recent progress in experimental studies has provided a wealth of information that can be used to gain understanding of the molecular mechanisms of myofascial pain syndrome. Only through improved understanding of the molecular and subcellular pathways behind this disorder can novel therapeutics be discovered. This improved comprehension might also help guide current treatment protocols for optimal benefit. However, many details of the signaling pathways involved remain yet unclear and further study is needed. Finally, the analysis presented here suggests that colchicine is a likely therapeutic that should be explored further as a treatment for myofascial pain.

## Glossary

ASIC: Acid-sensing ion channel with isoforms ASIC1, ASIC2, ASIC3, and ASIC4
ATP: Adenosine triphosphate
CGRP: Calcitonin gene related peptide
EPP: End plate potential
ETC: Electron transport chain
ERK: Extracellular signal-related kinase
FAK: Focal adhesion kinase
gp91^phox^: Another name for NOX2
H_2_O_2_: Hydrogen peroxide
MEPP: Miniature end plate potential
NADPH: Nicotinamide adenine dinucleotide phosphate
NOX: NADPH oxidase with isoforms NOX2 and NOX4
p22: Regulatory subunits of NOX2
p47^phox^: Regulatory subunits of NOX2
p67^phox^: Regulatory subunits of NOX2
p40^phox^: Regulatory subunits of NOX2
PLA2: Phospholipase A2
Rac1: Ras-related C3 botulinum toxin substrate 1 acting as regulatory subunits of NOX2
Rho: A small prokaryotic protein involved in the termination of transcription
ROS: Reactive oxygen species
RyR: Ryanodine receptor
RyR1: Ryanodine receptor type 1
SOD: Superoxide dismutase
TRP: Transient receptor potential channel with isoforms TRPM2, TRPM7, TRPC5, TRPV1, TRPV2, TRPV3, TRPV4, TRPA1, and TRPM8
XO: Xanthine oxidase
X-ROS: Stretch activated ROS production by NOX2 to potential calcium signaling.
